# Reported incidence of fever for under-5 children in Zambia: a longitudinal study

**DOI:** 10.1186/s13690-015-0097-5

**Published:** 2015-11-30

**Authors:** Benson M. Hamooya, Gershom Chongwe, Lungowe Sitali, Hikabasa Halwindi

**Affiliations:** Ministry of Health, P.O Box 30205, Lusaka, Zambia; Department of Public Health, The University of Zambia, School of Medicine, P.O. Box 50110, Lusaka, Zambia; Department of Biomedical Sciences, The University of Zambia, School of Medicine, P.O Box 50110, Lusaka, Zambia

**Keywords:** Fever, Malaria, Incidence, Under five children, Longitudinal study

## Abstract

**Background:**

Childhood fever is the most common clinical sign of *Plasmodium falciparum* infection. It is used as a measure of burden of the disease and the effectiveness of control programs for malaria. This study aimed to determine the incidence of fever in under-5 children of Magoye and Chivuna rural areas of Mazabuka district, Zambia.

**Methods:**

Incidence of fever was evaluated longitudinally over a period of 16 months (July 2006 and November 2007) among children aged 12–59 months in Magoye and Chivuna rural communities. The data was collected for a study on community directed treatment of soil-transmitted helminth infections in under-five children. Data from caretakers of 1221 children were collected using a structured interviewer-administered questionnaire. Cox proportion hazard regression was used to determine predictors of multiple episodes of fever and Kaplan-Meier survival curves was used to compare survival between two groups.

**Results:**

A total of 1221 under-5 children [median age 32 months; IQR 12–58] participated in the study and 696 (57 %) were from Magoye and 525 (43 %) from Chivuna. The incidence rate of fever was 162.4 per month per 1000 children for the 16 months period. The proportion of fever was not statistically related to children’ age [*p* = 0.779] and sex [*p* = 0.546]. Predictors of multiple episodes of fever were: age (37–48 vs. 12–24 months) [HR 0.81; 95 % CI 0.67, 0.98; *p* = 0.030]; location (Chivuna vs. Magoye) [HR 1.35; 95 % CI 1.17, 1.56; *p* < 0.001]; and season (dry vs. rainy) [HR 0.17; 95 % CI 0.12, 0.23; *p* < 0.001].

**Conclusion:**

The study has shown that the incidence of fever was high in the study areas. Febrile illnesses like malaria still have a significant effect on the health of under-5 children in the study population. There still exists the need for interventions aimed at reducing the incidence of fever in under five children, more especially in rural areas.

## Background

Malaria is a parasitic disease that is transmitted to humans by a female anopheles mosquito infected with one of the five species (*falciparum, vivax, ovale, malariae and knowlesi*) from the genus *plasmodium* [1]. *Plasmodium falciparum* is the most prevalent species in Africa. Usually the symptoms of infection appear 9 to 14 days after a bite from an infectious mosquito and typically include fever, headache, vomiting, and other flu-like symptoms. If not treated, malaria can be dangerous to life. Malaria is, therefore, mentioned as the commonest cause of fevers [2–6] and in many malaria endemic areas, fever has been used as a proxy for malaria, although the cause might be different [4]. Childhood fever is the most common clinical sign of *P. falciparum* infection and used as a measure of public health burden of the disease, and the effectiveness of the programs aimed at preventing malaria [7].

Globally, 3.4 billion people are at risk of malaria, with 1.2 billion at high risk (>one case per 1000 population) [8]. In 2013, World Health Organization (WHO) estimated 198 million cases of malaria to have occurred globally, out of which 584 000 deaths were estimated. The majority of cases (82 %) and deaths (90 %) estimated occurred in WHO African region, in which under-5 years accounted for 78 % of all the deaths that occurred [9]. From the year 2000, more than half of the countries that had ongoing malaria transmission have recorded decreases in the incidence of confirmed and reported cases of malaria. This was because of the scale-up of malaria interventions between 2000 and 2013, which led to the reduction of the incidence rates of the disease by 30 % globally, and by 34 % in Africa [8]. Between 2000 and 2013, mortality rates of malaria decreased by 47 % globally and by 54 % in Africa. In under-5 children the decline in mortality rate was by 53 % globally and by 58 % in Africa [8]. There is an estimated decrease of malaria incidence by 2015 of 56 and 63 % in all age groups and children under-5 years of age respectively if the annual rates of decrease is maintained [10].

In Zambia, malaria is one of the leading causes of morbidity and mortality among children under the age of five [11]. It accounts for up to 40 % of all infant mortality of which 15–20 % of deaths happen in children under-5 years of age. Over the past five years, Zambia has significantly intensified the efforts against malaria by initiating and scaling up of internationally accepted prevention and control strategies [12]. Despite that, febrile illnesses more especially caused by malaria remains a public health problem in our rural areas. The aim of this study was to determine the incidence of reported fever in under-5 children of Chivuna and Magoye rural communities.

## Methods

### Study design and population

This longitudinal study determined the incidence of fever in under-5 children of Chivuna and Magoye rural communities in Mazabuka district, Zambia. About 70 % of the population in Mazabuka district is rural, consisting of peasants who depend on subsistence farming as their source of food and income. Their major crops are maize and cotton. Working on part-time or full-time basis in the nearby commercial farms is also a common occupation. Formal education levels are low, rarely going beyond 7^th^ grade. The majority of the settlers in the area are the Tonga speaking people; the largest tribe in the southern part of Zambia. Geographically, the two areas have generally flat land with numerous small rivers, but Chivuna is mountainous in few areas.

### Data collection

The data on 1221 under-5 children was collected by ten (10) trained field assistants using structured questionnaires from their caretakers during the period of July 2006 and November 2007 on a monthly basis. The main categories of questions in the questionnaire were developed based on what previous studies had used and reported, and included general social-demographic factors, and experience of fever in the preceding one month. The piloting of questionnaire and field assistants was done on 5 % of the total sample size. The caretakers determined the presence or absence of fever by only touching the body. The fever was used as a proxy measure for malaria.

Ethical approval for the study was obtained from Excellence in Research Ethics and Science Converge Institutional Review Board, Zambia (Ref. No. 2014-May-033).

### Data analysis

Data was analysed using STATA (STATACORP, version 12, College Station, Texas, USA). Chi-square test was used to compare proportions of more than two groups. Proportions of two groups were compared using Two- sample test of proportions. Two-sample Wilcoxon rank sum test (Mann –Whitney *U* test) was used to ascertain the significance of two groups’ median values. Survival between groups was compared by comparing the survival functions using the Kaplan-Meier survival curves. To test for significance, Log-Rank Test was used. The survival function analysis was able to deal with censoring. Univariate and multivariable Cox proportional hazard regression was used to assess predictors associated with multiple episodes of fever. Confidence interval (CI) of 95 % and a 5 % level of significance was used to assess statistical significance.

## Results

### Basic characteristics of study participants

The total sample consisted of 1221 children aged between 12 and 59. These participants were followed-up on a monthly basis for reported fever outcome. The median age of the children was 32 months (range 12–58) and mean duration of follow-up of 9.6 months (standard deviation 4.6).

Most of the children were from Magoye 696 (57 %) while 543 (43 %) were from Chivuna. There was no statistical difference (*p* = 0.234) in the distribution of different age groups in the two study areas. There was no statistical difference (*p* = 0.251) in the overall distribution of males and females in the two study areas as shown in Table 1.

**Table 1:** Magoye had a significantly higher probability of survival of under-5 children from fever compared to Chivuna (*p* < 0.001) as shown in Fig 2

	Study site	n (%)	
Parameter	Magoye, 696 (57)	Chivuna, 525 (43)	*p*-value for chi-square test
Age (months)			
12–24	155 (30)	122 (32)	0.234
25–36	151 (29)	96 (26)	
37–48	139 (26)	86 (23)	
49–60	79 (15)	70 (19)	
Sex			
Male	304 (50)	233 (54)	0.251
Female	303 (50)	201 (46)	

*N* number

### Incidence of fever

The overall incidence rate of fever in the study sites was 162.4 per month per 1000 under-5 children during the period between July 2006 and November 2007. However, the incidence rate was lower in Magoye compared to Chivuna. The monthly incidence rate of fever in Magoye was at 143.2 per 1000 under-5 children whereas in Chivuna it was at 193.4 per 1000 under-5 children per month, the difference was statistically significant (95 % CI 131.9, 155.4 and 176.9, 211.5 respectively). The median survival time was four months, interquartile range (IQR) (2, 9) and the total person time at risk was at 6489 months. Incidence rate of fever stratified by gender showed not significantly difference between males and females [164.7 (95 % CI 150.4, 180.3) and 159.8 (95 % CI 145.3, 175.7) per 1000 children, respectively], even though it was slightly higher in males. Incidence rate of fever was significantly higher in the rainy season (November to April) at 218.7 (95 % CI 201.8, 237.0) per month per 1000 children compared to the dry season (May to October), which had 122.2 (95 % CI 111.5, 133.8). Children aged between 12–24 months had a higher incidence rate of fever (180.0 per month per 1000 children) as compared to the ones aged 37–48 months whose incidence rate was at 147.3 per month per 1000 children for the period of 16 months. This is shown in Table 2 below.

**Table 2:** Incidence rate of fever by Location, Sex, Season and Age (months) over 16 months follow-up in Magoye and Chivuna rural parts of Zambia

Characteristics	Number of fevers	Total person-months at risk	Incidence rate^*^1000 (95 % CI)
Overall	1054	6489	162.4 (152.9, 172.5)
location			
Magoye	573	4002	143.2 (131.9, 155.4)
Chivuna	481	2487	193.4 (176.9, 211.5)
Sex			
Male	470	2854	164.7 (150.4, 180.3)
Female	426	2666	159.8 (145.3, 175.7)
Season			
Rainy	592	2707	218.7 (201.8, 237.0)
Dry	462	3782	122.2 (111.5, 133.8)
Age (months)			
12–24	241	1339	180.0 (158.6, 204.2)
25–36	212	1305	162.5 (142.0, 185.9)
37–48	191	1297	147.3 (127.8, 169.7)
49–60	137	717	191.1 (161.6, 225.9)

*CI* confidence interval

### Univariate analysis (chi-square)

The results of univariate analysis to assess for correlation between individual explanatory characteristics and development of fever are given in Table 3. A total of 362 (29.6 %) participants were reported to have fever.

**Table 3:** Basic characteristics of the study participants sorted according to outcome

Factors	% (95 % CI)		
	Fever	No fever	P-value
	*n* = 362 (29.6 %)	*n* = 859 (70.4 %)	
Age (months)			
12–24	33 (27, 39)	30 (26, 34)	0.779|^a^
25–36	26 (21, 32)	28 (24, 32)	
37–48	25 (20, 31)	25 (22, 29)	
49–60	15 (11, 20)	17 (14, 20)	
Location			
Magoye	52 (47, 57)	59 (56, 62)	0.028|^a*^
Chivuna	48 (43, 53)	41 (38, 44)	
Sex^c^			
Male	53 (47, 59)	51 (47, 55)	0.546|^a^
Female	47 (41, 53)	49 (45, 53)	
ITN ownership^c^			
Yes	36 (31, 41)	N/A	<0.0001|^b*^
No	64 (59, 69)	N/A	
Who slept under ITN^c^			
Under-five children	91 (84, 96)	N/A	<0.0001|^b*^
Others	9 (4, 16)	N/A	
Seasons			
Rainy	33 (28, 38)	35 (32, 38)	0.457|^a^
Dry	67 (62, 72)	65 (62, 68)	

*N* number of participants

^a^ Chi-square test.

^b^ Two- sample test of proportions. N/A means Not Applicable; questions were only for respondents whose children had fever

^c^Percentages worked on less numbers from the overall due to missing values

^*^Significant finding (*p* < 0.05)

The median age did not differ significantly between children that had fever and children that did not (median 32 vs. 33 months; *P* = 0.504). The proportion of fever cases decreased with respect to an increase in the age (12–24, 25–36, 37–48 and 49–60) of a child (33 %, 26 %, 25 % and 15 %, respectively), although it was not statistically significant (*p* = 0.779). The proportion of children with fever was significantly higher (64 %) among under-five children whose households did not own insecticide treated nets (ITNs) compared to households with insecticide treated nets (36 %), with *p* < 0.001. Furthermore, an under-five child was significantly more likely to sleep under an ITN (91 %) compared to any other person in the household (9 %), *p* < 0.0001.

### Predictors of multiple episodes of fever

Univariate analysis (Table 4) showed that children aged between 37–48 months had a significant protective effect against fever by 19 % (1–0.81)*100 (HR 0.81; 95 % CI 0.67, 0.98; *p* = 0.033) in comparison to those aged 12–24 months. There was no statistical difference in fever episodes in terms of sex (HR 0.97; 95 % CI 0.85, 1.11; *p* = 0.688). There was also no statistical difference in fever episodes between households with ITNs vs. no ITNs (HR 1.04; 95 % CI 0.91, 1.19; *p* = 0.601). Dry season had a significant protective effect against fever episodes by 80 % (HR 0.20; 95 % CI 0.16, 0.25; *p* < 0.001) when compared to rainy season. A child residing in Chivuna area was at 1.34 times risk of having a fever episode compared to one in Magoye area (HR 1.34; 95 % CI 1.19, 1.52; *p* < 0.001). In terms of health seeking, there was 1.15 likelihood of seeking treatment from community health workers than from a Health facility, but the findings were not statistically significant (*p* = 0.158). During adjusted analysis (Table 4), season was still significantly associated (*p* < 0.001) with fever episodes and all other variables were statistically not significant. Hence, the best predictors’ model (Table 5), showed that age (12–24 vs. 37–48), location and season were the predictors that were significantly associated with multiple episodes of fever.

**Table 4:** Predictors of multiple episodes of fever (Univariate and Multivariate analysis)

Variables	Univariate HR (95 % CI)	*P*-value	Adjusted HR (95 % CI)	*P*-value
Age (months)				
12–24	1.00	1.00	1.00	1.00
25–36	0.91 (0.75, 1.09)	0.294	0.96 (0.76, 1.20)	0.696
37–48	0.81 (0.67, 0.98)	0.033^*^	0.84 (0.67, 1.06)	0.151
49–60	1.05 (0.85, 1.30)	0.624	1.00 (0.78, 1.28)	0.997
Sex				
Male	1.00	1.00	1.00	1.00
Female	0.97 (0.85, 1.11)	0.688	0.97 (0.82, 1.15)	0.705
Location				
Magoye	1.00	1.00	1.00	1.00
Chivuna	1.34 (1.19, 1.52)	<0.001^*^	1.14 (0.93, 1.390	0.208
ITN ownership				
Yes	1.00	1.00	1.00	1.00
No	1.04 (0.90, 1.19)	0.601	1.19 (0.97, 1.47)	0.088
Season				
Rainy	1.00	1.00	1.00	1.00
Dry	0.20 (0.16, 0.25)	<0.001^*^	0.21 (0.15, 0.30)	<0.001^*^
Source of treatment				
Health facility	1.00	1.00	1.00	1.00
Comm. Health Workers	1.15 (0.95, 1.39)	0.158	1.12 (0.86, 1.45)	0.392
Other sources	0.85 (0.69, 1.05)	0.129	0.84 (0.66, 1.09)	0.190

*HR* hazard ratio, *CI* confidence interval. Other sources (Friend, Relatives, Traditional healers or Spiritualists). *Comm* community

^*^Significant finding (*p* < 0.05)

**Table 5:** Adjusted predictors of multiple episodes of fever (hazard ratio) from the best model that fits the data well

Predictors of fever episodes	HR (95 % CI)	*p*-value
Age (months)		
12–24	1.00	1.00
25–36	0.88 (0.73, 1.06)	0.173
37–48	0.81 (0.67, 0.98)	0.030^*^
49–60	1.02 (0.83, 1.26)	0.862
Location		
Magoye	1.00	1.00
Chivuna	1.35 (1.17, 1.56)	<0.001^*^
Season		
Rainy	1.00	1.00
Dry	0.17 (0.12, 0.23)	<0.001^*^

*HR* hazard ratio, *CI* confidence interval

^*^Significant finding (*p* < 0.05)

### Kaplan-Meier (K-M) survival estimates

**Fig. 1:**
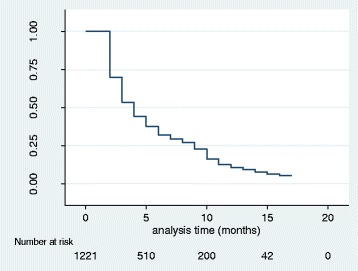
K-M Survival Estimate; Probability of not having Fever for all Participants

**Fig. 2:**
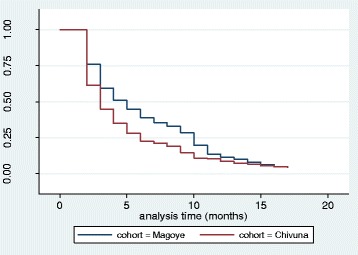
K-M Survival Estimates; Probability of not having Fever by Study Site

**Fig. 3:**
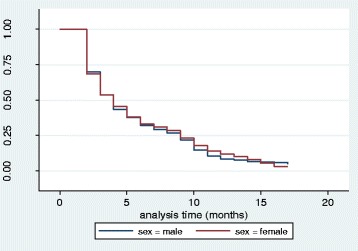
Kaplan-M Survival Estimates; Probability of not having Fever by Sex

The overall survival, number at risk and events experienced at each time interval during the study period is shown in Fig. 1. At the beginning of the study, 1221 under-5 children were recruited for a 16 months follow-up for fever outcome. The probability of survival from fever was reduced to about 5 % by the end of the study. Hence, an under-5 child in both Magoye and Chivuna rural communities had a probability of about 95 % of having fever in a period of 16 months. Magoye had a significantly higher probability of survival of under-5 children from fever compared to Chivuna (*p* < 0.001) as shown in Fig. 2. Figure 3 shows that, there was no significant difference (*p* = 0.6474) in the probability of survival from fever between males and females in the study.

## Discussion

The incidence of fever in under-five children in some rural parts of Zambia was still a huge challenge as it was revealed by this study. Meanwhile malaria has continued to be a major cause of morbidity and mortality in Zambia [13]. The study determined the reported incidence of fever in under-5 children of Magoye and Chivuna rural communities of Mazabuka district, Southern province, Zambia. Furthermore, the study explored predictors of multiple episodes of fever.

The overall incidence rate of fever in the study participants was high in 16 months period. However, there was some variation when stratified by other variables. In this study, the incidence rate of fever in the rainy season was higher than in the dry season. This compares well with other studies which had similar findings [14, 15]. The high incidence of fever in the rainy season is probably due to higher rate of transmission of malaria infection during this season [16]. In many areas where malaria is endemic, transmission is seasonal, with the peak during and just after the rainy season [17]. This is because fever is one of the first major symptoms of malaria [8].

In Chivuna, there were more episodes of fever cases as compared to Magoye. The difference could have been impacted by a community intervention [Health facility (HF) + Community directed treatment (ComDT)] that was taking place in Chivuna rural community at the time the data was being collected, Magoye was a control (HF) for that community study. The findings are similar to the study conducted in Burkina Faso [18], where the number of malaria episodes treated in the intervention arm [HF + Home Management of Malaria (HMM)] was much higher than in the control arm (HF). This is because, when the treatment option is close to home, caretakers tend to make use of it and hence the increase in the number of fever or malaria episodes being treated. Other probable reasons could be due to the availability several water bodies in Chivuna and it being more rural than Magoye. Magoye is located along a highway as such it could be possible that accessibility to preventative measures is easier than in Chivuna.

Gender was not a significant predictor of fever in under-five children. This finding is in line with the study that was conducted in rural Thatta, Pakistan [19]. According to the available evidence, it is suggested that given an equal exposure, male and female are equally vulnerable to malaria infection [20]. However, the age of a child, specifically those aged 12–24 and 37–48 months was a significant predictor of multiple episodes of fever, in which the youngest group had the highest burden of fever. This is similar to findings in other studies [15, 19, 21, 22], in which it was found that, children aged one to two years had the highest proportion of fever or malaria burden. This is likely to be the result of inadequate immunity which results in rapid increase in the parasite count and development of complications [23]. The infants in the first two months of life are protected by passive immunity offered by maternal antibodies and as a child grow from 2 years and above, they start to develop immunity. The study also revealed that, the incidence rate of fever in those aged 12–24 months was higher as compared to the children aged 37–48 months.

The households without insecticide treated nets (ITNs) had more cases of fever as compared to those households that owned ITNs. This result is similar to that of earlier studies [24–26]. According to Atieli et al., [27] households who own ITNs and make use of them are protected from mosquito bites , thereby having reduced episodes of fever or malaria infection. However, adjusted analysis in our study revealed that ITN ownership was not found to be a significant predictor of multiple episodes of fever. This could have been probably due to ownership not always translating into usage, although a study in Tanzania [28], revealed that there was no significant difference in ownership and usage of ITNs. However, among the households that owned ITNs, the findings were that more of the under-5 children slept under the ITNs than any other person in a particular household, as also reported by studies in Nigeria, Africa and Ethiopia [29–31]. In most cases, the youngest children are given more care than older ones in a household; this could explain the probable reason why more under-5 children slept under the ITNs.

### Limitation of the study

Several limitations may arise because of a data whose initial purpose of collection was different. The aim of the parent study was to determine the effectiveness of adding community directed treatment on health facility (HF + ComDT) with regard to treatment of soil-transmitted helminth infections and care-seeking behaviour compared to the model that had only health facility (HF). However, during that process longitudinal data on childhood fever was collected which this present study analysed. The data was also collected in 2007; hence, the need to do a current study to see if things have changed.

Perceived fevers in under-5 children which were reported over the period of 16 months by their caretakers to determine the incidence of fever may have been defined wrongly as any poor health rather than a biologically equivalent and clinically increased body temperature [32]. Not all fevers are malaria [33, 34], as this study used fever as a proxy for malaria. The data also could have been subject to recall bias as the caretakers were made to remember whether the child had fever in the previous month. However, a review study in Tanzania showed that, caretakers had generally a good biomedical understanding of febrile illnesses in terms of both types and symptoms [28]. Despite these limitations, in most malaria endemic countries with limited resources, fever in children is still used as a proxy for malaria.

#### Recommendations

The following are the recommendations from this study: To enhance the control and preventive programmes aimed at reducing fever or malaria incidence, like ITN utilization especially in the rainy season. There is need to conduct a longitudinal study specifically on the confirmed cases of malaria in under-5 children so as to have a more clearly picture with regards to incidence rate and care-seeking behaviour. The need to conduct a recent study as this study was conducted in 2006/2007; a lot might have changed.

## Conclusion

The study was able to show the incidence of fever in under-5 children of Magoye and Chivuna rural parts of Zambia. For the period of 16 months, the incidence rate of fever was high among under-5 children and they had a minimal probability of not having fever. Hence, the prevalence of fever in under-5 of Magoye and Chivuna rural parts of Mazabuka district is still a public health problem. This could have been a contributing factor to under-5 mortality. Hence, the need for examination of the control and preventive measures and reinforcement of the programmes aimed at reducing the incidence of fever or malaria in the communities.

## References

[1] Malaria report, “Malaria Disease Report. The FasterCures Philanthropy Advisory Service FasterCures / The Center for Accelerating Medical Solutions 1101 New York Ave. St., NW, #620 Washington, D.C. 20005 (202) 336–8900 www.philanthropyadvisoryservice.org,” 2009. [Online]. Available: http://www.fastercures.org/assets/Uploads/MalariaDiseaseReport.pdf. [Accessed: 17-Apr-2015].

[2] Molyneux CS, Mung’ala-Odera V, Harpham T, Snow RW (1999). Maternal responses to childhood fevers: a comparison of rural and urban residents in coastal Kenya. Trop Med Int Health.

[3] Tarimo DS, Lwihula GK, Minjas JN, Bygbjerg IC (2000). Mothers’ perceptions and knowledge on childhood malaria in the holendemic Kibaha district, Tanzania: implications for malaria control and the IMCI strategy. Trop Med Int Health TM IH.

[4] Uzochukwu BSC, Onwujekwe EO, Onoka CA, Ughasoro MD (2008). “Rural–urban Differences in Maternal Responses to Childhood Fever in South East Nigeria,”. PLoS One.

[5] Oyekale AS (2015). Assessment of Malawian Mothers’ Malaria Knowledge, Healthcare Preferences and Timeliness of Seeking Fever Treatments for Children Under Five. Int J Environ Res Public Health.

[6] Shayo EH, Rumisha SF, Mlozi MRS, Bwana VM, Mayala BK, Malima RC, Mlacha T, Mboera LEG (2015). Social determinants of malaria and health care seeking patterns among rice farming and pastoral communities in Kilosa District in central Tanzania. Acta Trop.

[7] Olotu A, Fegan G, Williams TN, Sasi P, Ogada E, Bauni E, Wambua J, Marsh K, Borrmann S, Bejon P (2010). “Defining Clinical Malaria: The Specificity and Incidence of Endpoints from Active and Passive Surveillance of Children in Rural Kenya. PLoS One.

[8] WHO Factsheet, “WHO | Factsheet on the World Malaria Report 2014,” WHO, 2014. [Online]. Available: http://www.who.int/malaria/media/world_malaria_report_2014/en/. [Accessed: 03-Mar-2015].

[9] World Malaria Report, “WHO | World Malaria Report,” WHO, 2014. [Online]. Available: http://www.who.int/malaria/publications/world_malaria_report/en/. [Accessed: 03-Mar-2015].

[10] World Malaria Report, “WHO | World Malaria Report 2013,” WHO, 2013. [Online]. Available: http://www.who.int/malaria/publications/world_malaria_report_2013/report/en/. [Accessed: 09-Mar-2014].

[11] Yeboah-Antwi K, Pilingana P, Macleod WB, Semrau K, Siazeele K, Hamainza B, Seidenberg P, Mazimba A, Sabin L, Kamholz K, Thea DM, Hamer DH (2010). “Community case management of fever due to malaria and pneumonia in children under five in Zambia: a cluster randomized controlled trial. PLoS Med.

[12] Malaria control centre/ministry of health, “Progress and impact of malaria control in Zambia at a glance: Roll back Malaria,” 2011. Available from: <http://www.rollbackmalaria.org/resources/progress-impact-series/country-reports> [Accessed 25 March 2014].

[13] MalariaCare, “Achieving universal diagnosis and appropriate treatment of malaria in Zambia,” MalariaCare, 2014. Available from: <http://malariacare.org/where-we-work/zambia-country-program/> [Accessed 6 March 2015].

[14] Ehrhardt S, Burchard GD, Mantel C, Cramer JP, Kaiser S, Kubo M, Otchwemah RN, Bienzle U, Mockenhaupt FP (2006). Malaria, Anemia, and Malnutrition in African Children—Defining Intervention Priorities. J Infect Dis.

[15] Ouédraogo A, Tiono AB, Diarra A, Sanon S, Yaro JB, Ouedraogo E, Bougouma EC, Soulama I, Gansané A, Ouedraogo A, Konate AT, Nebie I, Watson NL, Sanza M, Dube TJT, Sirima SB (2013). “Malaria Morbidity in High and Seasonal Malaria Transmission Area of Burkina Faso,”. PLoS One.

[16] Africa IRSZambia (2014). “Zambia- Africa Indoor Residual Spray Project,”. Africa IRS.

[17] WHO, “WHO fact sheet on malaria providing key facts, definition, information on transmission, symptoms, who is at risk, diagnosis, treatment, prevention, insecticide resistance, surveillance, elimination, vaccines and WHO response.,” WHO, 2015. [Online]. Available: http://www.who.int/mediacentre/factsheets/fs094/en/. [Accessed: 19-May-2015].

[18] Tiono AB, Kabore Y, Traore A, Convelbo N, Pagnoni F, Sirima SB (2008). Implementation of Home based management of malaria in children reduces the work load for peripheral health facilities in a rural district of Burkina Faso. Malar J.

[19] Nuruddin R, Hadden WC, Petersen MR, Lim MK (2009). Does child gender determine household decision for health care in rural Thatta, Pakistan?. J Public Health.

[20] WHO, “WHO | Gender, health and malaria,” *WHO*, Jun-2007. [Online]. Available: http://www.who.int/gender/documents/malaria/gender_malaria_leaflet/en/. [Accessed: 28-Jan-2015].

[21] Saúte F, Aponte J, Almeda J, Ascaso C, Vaz N, Dgedge M, Alonso P (2003). Malaria in southern Mozambique: incidence of clinical malaria in children living in a rural community in Manhiça district. Trans R Soc Trop Med Hyg.

[22] Owusu-Agyei S, Asante KP, Adjuik M, Adjei G, Awini E, Adams M, Newton S, Dosoo D, Dery D (2009). A. Agyeman-Budu, and others, “Epidemiology of malaria in the forest-savanna transitional zone of Ghana,”. Malar J.

[23] Malaria in Children report, “Malaria in Children | Malaria Site,” 2015. Available from: <http://www.malariasite.com/malaria-children/> [Accessed 15 April 2015].

[24] Holtz TH, Marum LH, Mkandala C, Chizani N, Roberts JM, Macheso A, Parise ME, Kachur SP (2002). Insecticide-treated bednet use, anaemia, and malaria parasitaemia in Blantyre District, Malawi. Trop Med Int Health TM IH.

[25] Koram KA, Owusu-Agyei S, Fryauff DJ, Anto F, Atuguba F, Hodgson A, Hoffman SL, Nkrumah FK (2003). Seasonal profiles of malaria infection, anaemia, and bednet use among age groups and communities in northern Ghana. Trop Med Int Health TM IH.

[26] Osuorah DC, Ezeudu CE, Onah SK, Anyabolu OT (2013). “Household bed net ownership and use among under-5 children in Nigeria.,”. Res Rep Trop Med.

[27] Atieli HE, Zhou G, Afrane Y, Lee M-C, Mwanzo I, Githeko AK, Yan G (2011). Insecticide-treated net (ITN) ownership, usage, and malaria transmission in the highlands of western Kenya. Parasit Vectors.

[28] Kassile T (2012). “Prevention and management of malaria in under-five children in Tanzania: a review,”. Tanzan J Health Res.

[29] Oresanya OB, Hoshen M, Sofola OT (2008). “Utilization of insecticide-treated nets by under-five children in Nigeria: Assessing progress towards the Abuja targets. Malar J.

[30] Noor AM, Kirui VC, Brooker SJ, Snow RW (2009). The use of insecticide treated nets by age: implications for universal coverage in Africa. BMC Public Health.

[31] Deressa W, Fentie G, Girma S, Reithinger R (2011). Ownership and use of insecticide-treated nets in Oromia and Amhara Regional States of Ethiopia two years after a nationwide campaign. Trop Med Int Health.

[32] Einterz EM, Bates ME (1997). “Fever in Africa: do patients know when they are hot?,”. Lancet.

[33] Mbonye AK, Lal S, Cundill B, Hansen KS, Clarke S, Magnussen P (2013). Treatment of fevers prior to introducing rapid diagnostic tests for malaria in registered drug shops in Uganda. Malar J.

[34] P. Brasseur, C. Raccurt, M. Badiane, M. Cisse, J.-F. Trape, and C. Sokhna, “[Changes in malaria prevalence and management of fevers from 2000 to 2012 in Casamance, Senegal.],” Bull. Soc. Pathol. Exot. 1990, Nov. 2014 [Epub ahead of print]. 10.1007/s13149-014-0404-3 25407334

